# Mediators and Theories of Change in Psychotherapy for Young People With Personality Disorders: A Systematic Review Protocol

**DOI:** 10.3389/fpsyg.2021.703095

**Published:** 2021-09-20

**Authors:** Jana Volkert, Svenja Taubner, Rasa Barkauskiene, Jose M. Mestre, Célia M. D. Sales, Vanessa Thiele, Andrea Saliba, Sonja Protić, Asta Adler, Sonia Conejo-Cerón, Dina Di Giacomo, Yianna Ioannou, Patricia Moreno-Peral, Filipa Mucha Vieira, Catarina Pinheiro Mota, Marija Raleva, Margarida Isabel Rangel Santos Henriques, Jan Ivar Røssberg, Stefanie J. Schmidt, Tjasa Stepisnik Perdih, Randi Ulberg, Erkki Heinonen

**Affiliations:** ^1^Department of Psychology, MSB Medical School Berlin, Berlin, Germany; ^2^Institute of Psychosocial Prevention, University of Heidelberg, Heidelberg, Germany; ^3^Institute of Psychology, Vilnius University, Vilnius, Lithuania; ^4^Department of Psychology, University of Cádiz, Cádiz, Spain; ^5^Faculty of Psychology and Education Science, University of Porto, Porto, Portugal; ^6^Center for Psychology, University of Porto, Porto, Portugal; ^7^Institute of Psychology, University of Kassel, Kassel, Germany; ^8^Mental Health Services Malta, University of Malta, Msida, Malta; ^9^Institute of Criminological and Sociological Research Belgrade, Belgrade, Serbia; ^10^Instituto de Investigación Biomédica de Malaga, Málaga, Spain; ^11^Department of Life, Health and Environmental Sciences, University of L’Aquila, L’Aquila, Italy; ^12^Department of Social Sciences, University of Nicosia, Nicosia, Cyprus; ^13^Center for Psychology, University of Trás-os-Montes and Alto Douro, Vila Real, Portugal; ^14^Department of Child and Adolescent Psychiatry, University Clinic Skopje, Skopje, North Macedonia; ^15^Institute of Clinical Medicine, University of Oslo, Oslo, Norway; ^16^Department of Clinical Psychology and Psychotherapy, University of Bern, Bern, Switzerland; ^17^School of Advanced Social Studies, Nova Gorica, Slovenia; ^18^Department of Psychiatry, Diakonhjemmet Hospital, Oslo, Norway; ^19^National Institute for Health and Welfare, Helsinki, Finland; ^20^Department of Psychology, University of Oslo, Oslo, Norway

**Keywords:** systematic review, personality disorder, young adult, adolescence, mediator, mechanism, psychotherapy, treatment

## Abstract

**Background:** Personality disorders (PDs) are a severe health issue already prevalent among adolescents and young adults. Early detection and intervention offer the opportunity to reduce disease burden and chronicity of symptoms and to enhance long-term functional outcomes. While psychological treatments for PDs have been shown to be effective for young people, the mediators and specific change mechanisms of treatment are still unclear.

**Aim:** As part of the “European Network of Individualized Psychotherapy Treatment of Young People with Mental Disorders” (TREATme), funded by the European Cooperation in Science and Technology (COST), we will conduct a systematic review to summarize the existing knowledge on mediators of treatment outcome and theories of change in psychotherapy for young people with personality disorders. In particular, we will evaluate whether mediators appear to be common or specific to particular age groups, treatment models, or outcome domains (e.g., psychosocial functioning, life quality, and adverse treatment effects).

**Method:** We will follow the reporting guidelines of the Preferred Reporting Items for Systematic Reviews and Meta-Analyses (PRISMA) statement recommendations. Electronic databases (PubMed and PsycINFO) have been systematically searched for prospective, longitudinal, and case–control designs of psychological treatment studies, which examine mediators published in English. Participants will be young people between 10 and 30years of age who suffer from subclinical personality symptoms or have a personality disorder diagnosis and receive an intervention that aims at preventing, ameliorating, and/or treating psychological problems.

**Results:** The results will be published in a peer-reviewed journal and at conference presentations and will be shared with relevant stakeholder groups. The data set will be made available to other research groups following recommendations of the open science initiative. Databases with the systematic search will be made openly available following open science initiatives. The review has been registered in PROSPERO (evaluation is pending, registration number ID 248959).

**Implications:** This review will deliver a comprehensive overview on the empirical basis to contribute to the further development of psychological treatments for young people with personality disorders.

## Introduction

Personality disorders (PDs) are a severe health issue already prevalent among adolescents and young adults. The cumulative lifetime prevalence of PDs increases from 15% at the age of 14 to 28% at the age of 33 (Johnson et al., 2008). Furthermore, a persistent PD in adolescence is associated with higher risks of anxiety and depression and predicts significantly poorer functioning and greater impairments in the mid-thirties (Skodol et al., 2007; Moran et al., 2016). For example, with regard to borderline personality disorder (BPD), prevalence rates in adolescents are similar to those in adult populations, ranging between 1 and 3% in the community and 33–49% in clinical samples (cf. Videler et al., 2019).

Fortunately, earlier assumptions that PDs would be essentially untreatable and diagnosing them would lead to early stigmatization have been largely repudiated (Clark, 2009; Kaess et al., 2014). Rather, providing a fast and accurate treatment in adolescence is seen as potentially reducing disease burden and chronicity of symptoms and enhancing long-term functional outcomes (Lambert et al., 2013).

Most of the studies investigating the effectiveness of treatment have examined BPD specific in adult populations. In particular, Dialectical Behavior Therapy (DBT; Linehan, 1987), Mentalization-Based Treatment (MBT; Bateman and Fonagy, 2010), Transference-Focused Psychotherapy (TFP; Yeomans et al., 2014), and Schema-Focused Therapy (SFT; Young et al., 2008) are specialized and effective treatments for people with BPD (Storebø et al., 2020). However, with regard to young people with PDs, there are only a few studies on the effectiveness of psychotherapeutic treatments, focusing virtually exclusively on BPD, which seem to be generally effective, although, follow-up measurements are missing (Wong et al., 2019). At the same time, Jørgensen et al. (2021) consider the current-evidence base on psychological therapies for adolescents with BPD as inconclusive and hampered by high risk of bias, attrition rates, and underpowered studies.

Given that effects of psychotherapy vary, it is important to better understand the mediators of positive and negative treatment outcomes, i.e., what leads to adjustment and well-being and what leads to adverse life trajectories (e.g., Moffitt, 2018). From the viewpoint of personality over the life course, adolescence and young adulthood are periods of relatively rapid and strong change (Caspi et al., 2005; Clark, 2009).

This development of personality is determined by multiple factors and influences a number of life domains and outcomes. Differences in the efficacy of treatments may partly be attributed to these age-specific developmental challenges. Intrapersonal developmental factors include biological and psychological changes, such as the process of identify formation and building of self-regulation capacities (Erikson, 1973; Lohaus et al., 2010; King et al., 2018). Interpersonal, societal, and environmental factors include school achievements or career developments, finding a partner and raising a family, and financial concerns. A recent systematic review found that individual factors (e.g., childhood temperament and comorbid psychopathology) and current relational experiences (e.g., being exposed to peer-related violence in friendships and in romantic relationships) were predictive of worse outcomes, namely, stability or increase in the levels of BPD symptoms (Skabeikyte and Barkauskiene, 2021). Accordingly, when treating young adults there is a special need to address these age-specific, individual and relational risk factors and challenges. Since specialized treatment for BPD does not show similar superiority in adolescents as in adults, understanding age-specific mechanisms of change are needed to increase efficacy of treatments.

With regard to adult patients with PDs, a recent study by Kramer et al. (2020) reviewed the processes of how patients with PDs improve in psychotherapy. They found that emotional change including regulation, awareness, and transformation; socio-cognitive change including mentalizing, meta-cognition, and interpersonal patterns; and an increase in the insight and change in defense mechanisms are associated with recovery in treatment for patients with PDs. Similarly, Keefe and DeRubeis (2019) analyzed the mechanisms, which are mostly pursued in psychotherapy and are considered to be underlying constructs of PDs: Attachment, mentalization, core beliefs, personality organization, and use of defense mechanisms were identified as personality constructs that have been primarily investigated.

In sum, the authors stress that the maturation of the defense mechanisms needs to temporally precede an improvement of symptoms and functionality of personality organization. With regard to changes in attachment and mentalization, there is some empirical evidence of associations with improvement in outcomes; however, no mediation effect has been found. In psychodynamic therapies, transference interpretations seem to be associated with better outcomes (Keefe and DeRubeis, 2019). Accordingly, the question arises whether these mechanisms can also be identified in psychotherapy for young people. Furthermore, since personality is developing relatively rapidly and in numerous domains during adolescence and young adulthood, it is not obvious that the mediators of treatment success (or non-success) are uniform across this time period.

Another question addressed in this review will be whether similar mediating factors can be identified throughout this developmental period. Moreover, a third and a fourth question arise as to whether the processes and mechanisms suggested by Keefe and DeRubeis (2019) and Kramer et al. (2020) may also be present in different kinds of psychotherapies, and also be relevant and useful specifically for young people with PD.

Furthermore, in light of the diagnostic challenges of PDs (Hopwood et al., 2018), there is increasing empirical support for conceptualizing personality and PDs (Tackett et al., 2009; Krueger et al., 2012; Sharp et al., 2015) on a continuum or continuums, such as in the hierarchical taxonomy model of psychopathology (HiTOP; Kotov et al., 2017) or in the different domains of the Research Domain Criteria (RDoC; Insel et al., 2010). The RDoC (Insel et al., 2010) consist of five domains, which describe functionality on a continuous spectrum between normal and abnormal for humans, animals, and *in vitro*. The five domains include negative valence, positive valence, cognitive systems, systems for social processes, arousal/regulatory systems, and sensorimotor systems. The Hierarchical Taxonomy of Psychopathology (HiTOP; Kotov et al., 2017) orders psychopathological syndromes and subtypes on the basis of observed covariation of symptoms. In this process, related symptoms are grouped together and symptoms are combined into spectra for reducing heterogeneity and comorbidity of disorders. Accordingly, it is interesting to investigate whether mediators of treatment align with the continuums proposed by the taxonomy, for example, in HiTOPs conceptualization of externalizing vs. internalizing vs. thought disorders, or with regard to specific functioning domains in RDoC domains, for example, domains of social processes (e.g., perception and understanding of self or others) or arousal and regulatory systems (e.g., arousal).

Currently, there are no systematic reviews available investigating how exactly psychotherapy works for young people with PD. For this reason, the aim of this systematic review is to summarize the existing knowledge on mediators and theories of change in psychotherapy for young people with personality disorders. In particular, based on the empirical data and questions outlined above, we will investigate:

1. Age-specific mediators.
2. Treatment-specific vs. non-treatment-specific mediators for personality disorders.
3. PD-specific vs. non-specific mediators.
4. Outcome-specific mediators, including adverse events, subclinical severity of personality disorder symptoms, and psychosocial functioning.

This review is carried out as part of the “European Network of Individualized Psychotherapy Treatment of Young People with Mental Disorders” (TREATme), funded by the European Cooperation in Science and Technology (COST).

## Materials and Methods

### Search Strategy and Selection Criteria

The population, intervention, comparison, outcome, and study design (PICOS; Page et al., 2021) was used to define the research question. The review followed the Preferred Reporting Items for Systematic Reviews and Meta-Analysis (PRISMA; Moher et al., 2009) and this protocol was written following the PRISMA for protocols guidelines (PRISMA-P; Shamseer et al., 2015) checklist.

We use two methods to identify studies for this systematic review. First, we search the databases PsycINFO and Medline within the timeframe between 01.01.1990 and 31.12.2020 using search terms related to psychotherapy, young people, mediators, and personality disorders. The searches will be re-run just before the final analyses and thereby further studies retrieved for inclusion. Second, we use the ancestry and descendant approach search reference lists and citing articles of included articles and other relevant studies. The study is registered with PROSPERO under the record ID 248959 (registration status: submitted). The full search string is available at https://www.crd.york.ac.uk/PROSPEROFILES/248959_STRATEGY_20210414.pdf.

#### Types of Studies

Studies from any geographical location, written in English and published from 1990 onward until December 31st, 2020 that meet predefined inclusion criteria, will be included in the review. Gray literature, such as theses, dissertations, or conference proceedings, will also be included. Studies will be included if they include statistical analysis of a mediator of psychotherapy outcome. This comprises (a) empirical quantitative studies following prospective, longitudinal, and case–control designs, which include (b) terms related to or describing mediators, and (c) include a psychosocial intervention and/or psychotherapeutic intervention or treatment or primary/secondary prevention.

#### Types of Participants

Studies with a primary participant sample of young people between the age of 10 and 30years, with a diagnosis of any personality disorder (according to Diagnostic and Statistical Manual of Mental Disorders, DSM or International Statistical Classification of Diseases and Related Health Problems, ICD criteria; World Health Organization, 2008; American Psychiatric Association, 2013) or who have impairments in personality functioning and receive a psychotherapeutic intervention for their personality impairments, including primary and secondary prevention programs. All comparators will be included as we will investigate mediators in all treatments.

#### Types of Interventions

Studies will be included if they report an intervention aimed at preventing, ameliorating, or treating personality disorders in young people by using psychosocial mechanisms and strategies in any setting (i.e., individual, family, group, inpatients, and Mental health). Interventions that are primarily biological or physiological will not be included. Interventions can include all types of psychotherapy: cognitive or cognitive behavioral, interpersonal, integrative, humanistic (such as emotion focused, supportive, and motivational interviewing), psychoeducation, psychodynamic, systemic, third-wave approaches (such as mindfulness-based therapies), and disorder-specific approaches like dialectic-behavioral, mentalization-based, schema-focused therapy, and transference-focused therapy. Studies including adjunct pharmacotherapy to a psychological intervention will also be included. As comparators or control conditions, any type of comparator, including a waitlist control group, will be included. An inclusion of a control group is not a necessary requirement for an inclusion in the review but will be assessed and reflected critically.

#### Type of Outcome Measures

We will include any type of outcome measure that is used in intervention studies for young adults with personality disorders. In particular, we will include measures assessing different outcome areas that are specifically relevant for patients with personality disorders, including diagnosis, symptom severity, adverse events, and psychosocial functioning. The main outcome measures will be the statistical mediation effects from the intervention condition (IV) to the personality disorder outcome (DV) through a proposed mediator. If meta-analytic aggregation of the results is feasible, the *p* values or the bootstrap CI of a (intervention to mediator) and b (mediator to outcome) effects will be considered.

#### Type of Mediators

These intervention studies need to operationalize and examine the purported mechanisms of change as a mediator. That is, the mechanisms of change, or how an intervention is leading to change, should be operationalized as a mediator. According to Kazdin (2007), a mediator is an intervening variable that may account (statistically) for the relationship between the independent and dependent variable. A change in the mediator must follow the onset of the independent variable and precede change in the dependent variable temporally. In this study, any type of mediator that meets criteria of Kazdin (2007) will be assessed. A particular focus will be on hypothesized PD-treatment-specific mediators (for example, mentalization).

### Data Screening and Extraction

Study selection will be carried out by a group of 20 experienced researchers divided into 10 pairs who will independently assess the eligibility of studies retrieved using the search strategy in two phases. Prior to the start of the first phase, the researcher group will develop and agree on adhering to a homogeneous screening and rating procedure. In a first step of the data inclusion process, study title and abstract will be screened for whether they potentially meet the inclusion criteria outlined above. In the second phase, each pair of reviewers will evaluate the full text of these potentially eligible studies to check if they meet the inclusion criteria. Disagreements will be discussed in pairs, and a third reviewer will be involved if consensus cannot be reached. Finally, a fourth independent reviewer will perform an additional quality control check by assessing the eligibility of every fifth excluded study. Disagreements at this stage will be solved through discussion with the original screening researcher pair.

A standardized form will be used for data extraction. Extracted information will include as: authors, country of study, study design and setting, study population, participant demographics and baseline characteristics, details of the intervention and control conditions, study methodology, outcomes and times of measurement, mediators, mediator measures and type of mediation analysis, and information on the assessment of risk of bias. Two review authors will extract information independently, discrepancies will be identified and resolved through discussion or with a third author where necessary. Data records will be managed using Microsoft Excel (2013). Currently, no standard form for evaluating mediation studies has been established. Therefore, studies will be assessed according to the criteria for identifying mediators of psychosocial interventions in research, such as summarized by Kazdin (2007) and Lemmens et al. (2016).

### Data Synthesis

We will provide a narrative synthesis of the findings from the included studies, with focus on the types of mediators that have been tested, types of psychosocial interventions that have been investigated, and personality disorders or personality functioning impairments of young people that have been treated. It will be examined if age-, PD-, treatment-, and outcome-specific mediators can be identified. Included studies can be grouped by either age and/or intervention type (e.g., cognitive behavioral therapy and schema therapy) or between-group vs. within-group mediation analysis. The grouping procedure will depend on the final sample of included studies in the review. Studies will be reviewed and discussed in the context of the statistical mediation criteria outlined above. Furthermore, we will explore the extent to which current studies of mediators and theories of change can be meaningfully grouped into proposed categories of RDoC (Insel et al., 2010) and the HiTOP (Kotov et al., 2017).

If statistical aggregation of data is possible, standardized mean and standardized variance or SD will be recorded for each study individually. Following the statistical method of Wolf et al. (2016), group differences and mediation effects at recorded measurement points will be calculated using the “bias corrected standardized mean difference” (Hedge’s g) for each study individually. If possible, the strength of the influence of the mediators will be ordered by studied mediator in comprehension of the treatment and control group, e.g., in a forest plot. To account for differences in methods and samples of primary studies (Hedges and Vevea, 1998), a random-effects model will be used. We expect that only a qualitative summary of the influence of different mediators will be possible due to limited data. However, if sufficient study data are available, we will aggregate standardized effect sizes of the studies using the same mediation paths with Hedge’s g. Analysis of heterogeneity will be conducted with Cochran’s Q-test (Cochran, 1954) or I^2^ which should be preferred when the sample sizes of the primary studies are small (Higgins et al., 2003). To check for publication bias effect sizes, variance, and sample size will be illustrated in the funnel plot. Finally, if there are enough studies including different personality disorders, subgroup analyses could be conducted using different diagnostic groups classified in the DSM or ICD or different levels of personality functioning impairments. Furthermore, subgroup analyses may also be possible for different age subgroups (e.g., 10–20 and 21–30years) and different types of treatment (e.g., CBT, Psychodynamic, MBT, and SFT).

#### Risk of Bias Assessment

The Mixed Methods Appraisal Tool (Hong et al., 2018) will be used to evaluate the overall study quality using a formal risk of bias assessment. This tool permits appraisal of the methodological quality of five categories of studies: qualitative research, randomized controlled trials, non-randomized studies, quantitative descriptive studies, and mixed methods studies. Additionally, for evaluating the quality of the evidence and risk of bias for statistical mediation in the included studies, the criteria from Magill et al. (2020) will be used.

## Discussion

This paper described the study protocol of a systematic review that will assess mediators and theories of change in psychological treatments for adolescents and young people with personality disorders. To the best of the authors’ knowledge, this is the first systematic review of its kind that will systematize the existing empirical knowledge about mediators of intervention studies for this population and provide implications of this knowledge for future mediator studies and treatment planning and outcomes. In particular, we will highlight whether (and what kind of) age-, treatment-, PD-, and outcome-specific factors have been derived and need to be addressed in future research.

Furthermore, we will link the systematized evidence with theoretical models of mechanisms of change of treatments for young people with personality disorders, in particular those outlined in previously published reviews on mediators of psychotherapy for adults with personality disorders (e.g., Keefe and DeRubeis, 2019; Kramer et al., 2020) and explore the extent to which current studies of mediators and theories of change can be meaningfully grouped into proposed categories of RDoC (Insel et al., 2010) and HiTOP (Kotov et al., 2017).

The strengths of this review include the involvement of a large multidisciplinary group of international researchers with long-standing accumulated experience that have worked on this topic in a well-established setting. Furthermore, the group has consulted international experts in the field to develop this protocol. A standardized quality assessment procedure will be carried out as well as a search update to ensure the completeness of the data set. Furthermore, the data set will be made available to other research groups following the recommendations of the open science initiative.

Limitations of this protocol include the use of broad inclusion criteria, in particular with regard to intervention types and study designs, which likely limits the possibility of causal conclusions. However, it may likely not be feasible to estimate aggregated effect sizes for the identified mediators due to the limited number of studies. As there is no generally accepted gold standard for mediation analysis, we expect much variance in the studies, which could lead to the results being inconclusive or inconsistent. In addition, analyzing both subclinical conditions and diagnosable disorders, as well as intervention and prevention studies, may also lead to less consistent results. Furthermore, conclusions on mechanisms of change will only be related to empirical quantitative studies as qualitative and theoretical studies are not included in this review. As the rater team consists of a large group, quality assessment of the methodology has to be strictly monitored.

In light of the severity of impairment associated with personality disorders, the prevention and intervention at an early age are very important and more insight about treatment mediators is urgently needed. This review will yield the opportunity to obtain a comprehensive overview on the empirical basis in order to contribute to the further development of psychological treatments for young adults with personality disorders.

## Author Contributions

ST, AS, EH, SP, JV, AA, RB, SC-C, DG, YI, JM, PM-P, FV, CM, MR, MIR, JR, SS, TP, RU, CS, and VT provided a substantial contribution to the conception and design of the work by developing the research question, the search string, and carrying out the stage 1 screening. JV, ST, and EH drafted the manuscript. RB, JM, CS, VT, AS, SP, AA, SC-C, DG, YI, PM-P, FV, CM, MR, MIR, JR, SS, TP, and RU corrected and approved the manuscript. RU coordinated the overall COST initiative. All authors agree to be accountable for all aspects of the work in ensuring that questions related to the accuracy or integrity of any part of the work are appropriately investigated and resolved. All authors contributed to the article and approved the submitted version.

## Funding

This review was conducted as a part of a project by the European Network on Individualized Psychotherapy Treatment of Young People With Mental Disorders (TREATme; CA16102) to establish a sustainable European multidisciplinary researcher network focusing on individualized psychotherapy for young people with mental disorders (www.cost.eu).

## Conflict of Interest

The authors declare that the research was conducted in the absence of any commercial or financial relationships that could be construed as a potential conflict of interest.

## Publisher’s Note

All claims expressed in this article are solely those of the authors and do not necessarily represent those of their affiliated organizations, or those of the publisher, the editors and the reviewers. Any product that may be evaluated in this article, or claim that may be made by its manufacturer, is not guaranteed or endorsed by the publisher.
